# Parental Hopes and Understandings of the Value of Prenatal Diagnostic Genomic Sequencing: A Qualitative Analysis

**DOI:** 10.3389/fgene.2022.883225

**Published:** 2022-07-18

**Authors:** Simon M. Outram, Julia E. H. Brown, Astrid N. Zamora, Nuriye Sahin-Hodoglugil, Sara L. Ackerman

**Affiliations:** Program in Bioethics, University of California, San Francisco, San Francisco, CA, United States

**Keywords:** prenatal, genomics, sequencing, interviews (qualitative), empowerment

## Abstract

**Objective:** To provide qualitative empirical data on parental expectations of diagnostic prenatal genomic sequencing and the value of the results to families.

**Methods:** We interviewed 15 families—mothers and/or fathers—who had had prenatal genomic sequencing about their expectations and their respective evaluations of the benefits of genomic sequencing.

**Results:** Families’ hopes for genetic sequencing clustered around three themes: hoping to identify the cause of the fetal anomaly in a terminated pregnancy; hopes for guidance as to the likely outcome of current pregnancy; and hopes for information to support future family planning. In addition, hopes were discussed in terms of the potential for results to be beneficial in acquiring greater knowledge, while at the same time recognizing that new knowledge may raise more questions. Assessment of the value of sequencing largely mirrored these expectations when positive results seen. Negative results can also be seen as valuable in ruling out a genetic cause and in providing certainty that families had done everything that they could to know about the cause of fetal demise.

**Conclusion:** It would appear that with guidance from genetic counsellors, families were largely able to navigate the many uncertainties of prenatal genomic sequencing and thus see themselves as benefitting from sequencing. However, support structures are essential to guide them through their expectations and interpretations of results to minimize possible harms. Engaging in the process of genomic sequencing was seen as beneficial in of itself to families who would otherwise be left without any options to seek diagnostic answers.

## Introduction

Technological innovations, falling costs, and the development of rapid exome testing technologies suggest that prenatal diagnostic genomic sequencing is highly likely to become part of standard clinical practice in the near future if fetal anomalies are detected through ultrasound or other methods (Drury et al., 2015; Pangalos et al., 2016; Quinlan-Jones et al., 2017; Harris et al., 2018; Mellis et al., 2018; Richardson and Ormond, 2018; Ferretti et al., 2019; Sullivan et al., 2019). Fetal anomalies are identified in 2%–5% of pregnancies, the cause of which may remain unknown following chromosomal microarray and karyotype diagnosis. The employment of prenatal whole exome sequencing to identify etiology has drawn particular attention given that the cost of sequencing may be lower than for whole genome sequencing and may provide quicker results. Given that many treatment options are time-sensitive in a prenatal context, the speed of the sequencing process of key importance in respect to its clinical utility (Best et al., 2018; Lord et al., 2019). Studies have provided a wide range of figures in respect to diagnostic yield, with considerable variation due to study design, the fetal anomaly identified, and population intake (Best et al., 2018; Petrovski et al., 2019). However, a recent meta-analysis by Mellis et al. (2022) concludes that “prenatal ES [exome sequencing] provides a diagnosis in an additional 31% of structurally abnormal fetuses when CMA [chromosomal microarray]/karyotype is non-diagnostic.”

The psychological impact of receiving a prenatal diagnostic test showing fetal anomaly has been studied extensively (Werner-Lin et al., 2016; Wilpers et al., 2017; Hodgson and McClaren, 2018; Teefey et al., 2020; Bardi et al., 2021). Finding a fetal anomaly is self-evidently unwelcome and has been shown to increase the likelihood of anxiety, stress, and depression both within the time of the pregnancy and postpartum. It is possible that the increased diagnostic yield (over and above microarray and karyotype testing) provided by whole exome sequencing may offer opportunities to families for whom fetal anomalies have been detected to understand the cause of these respective anomalies and thus reduce stress. Clinical assessments, based upon the results of genomic sequencing, may also inform pregnancy management, help families prepare for the future after their child is born, and identify the risk of recurring issues in any future pregnancies. Conversely, the relatively low diagnostic yield for prenatal sequencing (with positive results of around 20%–30%) means that in most cases families will not receive a definitive genetic etiology. Moreover, even in cases where positive results are seen, care or treatment options are often limited (Quinlan-Jones et al., 2017; Abou Tayoun et al., 2018; Best et al., 2018; Horn and Parker, 2018; Mellis et al., 2018; Richardson and Ormond, 2018; Sullivan et al., 2019).

While both pediatric and prenatal genomic testing share commonalities in respect to the complexity of interpretation, prenatal genomic sequencing is likely to more challenging for clinicians to interpret and thus more difficult to clinicians and genomic counsellors to provide specific guidance to families. Fundamentally, the problem is that the phenotypic features of concern are not yet possible to observe within the fetus. As Horn and Parker state within the prenatal context, it is exceptionally difficult to “determine whether a variant will affect the resulting child if the pregnancy were to be continued.” Indeed, the extrapolation of prenatal genomic sequencing testing findings to make informed clinical decisions (including treatment, termination, and early screening for future pregnancy) is based upon knowledge from postnatally-derived (adult and minors) classifications found in ClinVar or the Human Gene Mutation Database along with emerging case studies in the literature (prenatal and postnatal). As several authors have noted development of a prenatal database might help to advance prenatal sequencing, but this is yet to be available (Drury et al., 2015; Aarabi et al., 2018; Abou Tayoun et al., 2018; Best et al., 2018; Horn and Parker, 2018; Mellis et al., 2018; Ferretti et al., 2019). Moreover, even if predictions about postnatal outcomes can be made with a strong degree of certainty, prenatal treatment options are often highly limited (Westerfield et al., 2015). These limitations are especially problematic given that decisions need to be made quickly during ongoing pregnancies.

### Ethical Concerns and the Need for Empirical Data

Ethical discussion of prenatal sequencing has frequently explored these counter-forces—the potential for genomic sequencing to provide strong indicators of treatment options with the likelihood of negative or uncertain results and limited treatment options. Much of the focus upon ethical concerns over prenatal testing is upon the potential for results to influence parental decisions as to whether to continue or end current pregnancies. Indeed, such life-changing decisions are all the more the problematic given the potential for results to be re-analyzed as more comparative data emerges (Horn and Parker, 2018; Mellis et al., 2018). More broadly, literature examines the degree to which families might be over-optimistic about the ability of sequencing to answer questions and perhaps underestimate the likelihood of an uncertainty future after results (Yurkiewicz et al., 2014; Kalynchuk et al., 2015; Chandler et al., 2018; Richardson and Ormond., 2018).

The contrast between the potential for genomic sequencing to inform families and enable some form of pregnancy management and the limitations of prenatal genomic sequencing make it important to talk with families, particularly mothers, about their experiences. Empirical data is only just emerging in respect to the experiences of families who have undertaken to have prenatal genomic sequencing (Kalynchuk et al., 2015; Quinlan-Jones et al., 2017; Richardson and Ormond, 2018; Wou et al., 2018; Plantinga et al., 2021; Talati et al., 2021). These studies have highlighted that sequencing may answer some questions but may also create difficult choices with respect to continuing a pregnancy. In addition, sequencing may identify likely postnatal outcomes needing considerable clinical intervention and high levels of risk for future pregnancy, potentially adding to parental stress. However, studies of families also indicate that despite limited options and uncertainties, for many the price of knowing is preferable to having little or no etiological information about the fetal anomaly identified (Quinlan-Jones et al., 2017; Plantinga et al., 2021). These studies are complimented by empirical data on the views and experiences of families who undertake microarray testing (Bernhardt et al., 2013; Hillman et al., 2013; Lewis et al., 2021) and those of healthcare providers and other experts on prenatal genomic testing and microarray testing (Shkedi-Rafid et al., 2016; Narayanan et al., 2018). Knowing the expectations of families and the value that they placed upon genomic sequencing enables clinicians and genetic counsellors to tailor their communication to provide neither false hope nor diminished enthusiasm.

In the following study, we talked with 15 families (mothers and/or fathers) about their experiences in respect to prenatal testing from an amniotic sample. This research was undertaken as a sub-study within a large whole exome sequencing research study undertaken at UCSF (details of the study are provided below). We talked to families about their expectations (and concerns) and what they thought about having had genomic sequencing. Families were interviewed twice, 2–4 weeks and 6 months after return of results. In doing so, the primary intention was to see whether the respective hopes of families for sequencing were matched to their evaluation of the benefits of sequencing. This paper is of relevance to clinicians, genetic counsellors, and policymakers who are broadly interested in knowing whether genomic sequencing provides a service that is helpful to families in their current pregnancies and in respect to family planning. More specifically, it is relevant to genetic counsellors and clinicians tasked with providing information to families who may be considering their options as to whether to have genetic sequencing following the detection of fetal anomalies.

## Methods

### Study Population Characteristics

The data presented in this paper was collected through interview and ethnographic observational analysis conducted within the University of California, San Francisco (UCSF) Program in Prenatal and Pediatric Genome Sequencing (P3EGS) study. The P3EGS study was approved by the IRB of USCF. A total 845 families (one or more parent) were enrolled in the study, of which 316 families were enrolled into the prenatal arm of the study. The P3EGS study is one site in a multi-sited research program, the Clinical Sequencing Evidence-Generating Research (CSER) Consortium. Parents of children with fetal anomalies the cause of which was not determined by amniocentesis were offered the opportunity to participate in the study free of charge. Participants underwent a lengthy process of genetic counselling at enrollment and during the return of results which highlighted the likelihood of negative results, limitations of any diagnosis in respect to the provision of treatment, and generally helped families prepare for the possibility of more uncertainty even after results are returned. Many of these sessions were observed by the ethnographic team (15 observations of enrollment and 32 observations of results sessions).

### Recruitment for Interviews

At the time of consent to the sequencing study families were informed verbally and in written documentation that they may be contacted by the ethnographic study to ask if they would be willing to be interviewed about their experiences. They were given the option to decline. This possibility was reiterated at the results session by genetic counsellors, clinical research coordinators, or members of ethnographic team when present (although in particularly stressful situations this offer of an interview was not made at the time of results).

### Sampling

Participants were selected for interviews based upon their results—positive, negative, or uncertain. The sampling was purposive, with the intention to over-sample families with positive and uncertain results (compared to the study population overall). Early interviews suggested that parents with negative results did not have much to say regarding the utility of genomic sequencing. As such, in order to maximize our qualitative understanding of the benefits (or otherwise) of sequencing results, it was important to over-sample positive and uncertain results.

### Data Collection

Of the 30 families contacted to request interviews 15 declined; those families who declined interviews were either passive decliners (no response to three requests by phone) or stated that they did not want to be interviewed. The dominant reason given for declining to be interviewed was lack of time.

Interviews were conducted between 2 and 4 weeks after results sessions and a follow-up interview was conducted 6 months after results sessions. Interviews generally lasted between 30 and 60 min with an average of 40 min. They included in-person, online, and phone interviews. Interviews were conducted between 2 April 2018, and 29 October 2020.

Interviews were arranged by phone and interviewees were fully informed that their decision to participate or not participate would in no manner influence ongoing clinical care. Potential interviewees (families who had agreed verbally to being asked about an interview) were called between 10 and 20 days after return of results to arrange interviews. In accordance with COREQ guidelines (Tong et al., 2007), interviewer credentials are provided in Table 1, below.

**TABLE 1:** Interviewer credentials.

Interviewer	Credentials	Occupation	Gender	Experience/Training
ID 1	PhD	Research specialist	Male	Multiple years of interviewing experience. Training/experience in ethnography and social scientific methods
ID 2	PhD/MPH	Associate professor	Female	Multiple years of interviewing. Training/experience in ethnography and social scientific methods
ID 3	BA	Research analyst	Female	Training and experience in interviewing. Currently in a genetic counseling master’s program. Fluent in spanish

The semi-structured interview guide included a wide range of topics including diagnostic journey, experience of enrollment and return of results, and subsequent understanding of results among others. Audio recordings of results sessions and interviews were professionally transcribed and were checked for quality and anonymity by the UCSF ethnographic study team.

### Analysis

The ethnographic and analytical approach taken is aligned to that Interpretative Description (Thompson Burdine et al., 2021) as employed in multiple studies exploring patient experiences for the purpose of developing educational tools or guidance to providers based upon these experiences. Interview analysis follows a data-driven themed analytical process as developed by Boyatzis (1998) and Braun and Clarke (2006) and further described by Deterding and Waters (2021) in respect to the employment of qualitative software to analyze interviews. Fieldnotes and interview transcripts were uploaded to Dedoose qualitative software allowing for multiple persons within the analysis team to share data. Thematic codes were developed in accordance with what was being learned through initial observations and interviews. These were reviewed and amended following their trial application within Dedoose. Upon finalization of codes and their application, each document (fieldnotes and interview transcripts) was reviewed by at least two members of the team for consistency. It is estimated that consistency between reviewers (overlapping coding using a blinded-coding methods) was approximately 75%–85%. Of the codes applied, the codes entitled “Expectations,” “Concerns,” and “Feelings about Results” were the most often applied to the following analysis but other findings outside of these codes were employed to add interpretive depth to the results presented below. Given the conceptual and methodological approach taken, a Kappa Coefficient was not produced for codes. Instead, coding overlap is provided as an indication of how themes were discussed among the team and consensus reached as to their interpretation and application. Lack of overlap was not necessarily something to be rectified, but instead was seen as an opportunity to widen the scope of interpretation for a particular code.

## Results

### Interviewee Population

A total of 15 families were included in the interview study. Table 2, below, provides key information on the 15 families interviewed. Of the 15 families interviewed, 6/15 [40%] received positive results, 4/15 received inconclusive results [27%], and 5/15 [33%] received negative results. Pregnancy status was also recorded with 8/15 [53%] families receiving results in respect to an ongoing pregnancy at time of enrollment and 7/15 families with terminated pregnancies. 8/15 [53%] families were members of an under-represented minority population. It is important to note that many of the participants had already ended their pregnancies—either spontaneously or electively—prior to enrollment in the study and/or prior to receiving genetic results (please see Table 2).

**TABLE 2:** Key interviewee characteristics.

FAM	Interviewed	Race/ethnicity	Classification of exome sequencing result	Pregnancy status at the time of results and interview
11	Mother	Hispanic	Negative	Terminated (prior to enrollment in study)
41	Mother	Asian/White	Inconclusive	Terminated (prior to enrollment in study)
86	Mother	White	Positive/*De Novo*	Terminated (prior to enrollment in study)
153	Mother & Father	Hispanic (Mother) & Asian (Father)	Inconclusive	Ongoing (at time of results and interview)
195	Mother	White	Positive/*De Novo*	Ongoing (at time of results and interview)/Deceased shortly after birth (prior to results and interview)
230	Mother & Father	Hispanic (Mother) & Hispanic (Father)	Positive/*De Novo*	Ongoing
260	Mother & Father	Hispanic/Asian (Mother) & Asian (Father)	Positive/*De Novo*	Ongoing
273	Mother	Asian	Inconclusive	Terminated (prior to enrollment in study)
309	Mother	White	Positive/*De Novo*	Ongoing at Results—Termination shortly after (prior to interview)
348	Mother	White	Negative	Terminated (prior to enrollment in study)
370	Mother	White	Negative	Ongoing
398	Mother	White	Positive/Maternal Inheritance	Ongoing
442	Mother	White	Negative	Terminated (prior to enrollment in study)
565	Mother & Father	Asian (Mother) & Asian (Father)	Negative	Ongoing
596	Mother	Hispanic	Inconclusive	Ongoing

### Thematic Analysis

Interviewee quotes have been organized to address the overarching question of whether the respective hopes of families were matched in their evaluation of the benefits of sequencing. Families’ hopes for genomic sequencing clustered around causality, likely outcomes, and implications for future pregnancy. Families also reflected upon how they entered sequencing with some concerns about how they might be left with difficult questions when results were returned. Their respective assessment of the value of prenatal genomic sequencing reflected these hopes and clustered around what they had learned in respect to the cause of fetal demise, implications of the results for outcomes in current pregnancy, and implications of results for future pregnancy. Interviewees also talked about upon the value of having done something to reduce uncertainty, regardless of test outcomes.

## Hopes for Genomic Sequencing

### Identifying Cause in Terminated Pregnancy

As might be expected when pregnancy has been ended, families wanted to find out what happened to cause the fetal anomaly and thus gain a form of closure. As one parent stated,

Like you could do whatever you need to do. We want to find out like what caused it. [0011/Negative Result/Termination]

This was sometimes combined with families wanting to know if the genetic variant that caused fetal demise had been passed down, as seen in the following excerpt,

MOTHER: I wanted to find out what happened. I wanted some answers about what happened to the fetus, and did it come from one of our genetic imprints, like “did we pass this on to the baby or was it an anomaly?” [0041/Positive Result/Termination]

### Likely Outcomes in Ongoing Pregnancy

In ongoing pregnancies with fetal complications, further information as to the likely outcome of the current pregnancy was of key importance, as seen below,

INTERVIEWER: What did you hope to learn from participating in this study?

MOTHER: If there was anything that—for lack of a better word—generally wrong with myself or my baby … She [the clinician] said that this study would give me, I think, 90% certainty that the baby was fine or not. That’s why I accepted to do the test. [0398/Positive Result/Ongoing]

In addition, as well as pregnancy outcome, it was hoped that sequencing might provide some indication as to postnatal care requirements,

MOTHER: So that was, I guess, what we were hoping is to see if there is anything else that we should be aware of in the future for us or for the baby. And so that is what we were kind of hoping to get out of it. And we did. [0565/Negative/Ongoing]

MOTHER: They would tell us what care we should have once she was born, knowing exactly what she had, and that they would give us much more information, like places where we could go after she was born to know exactly what to do. [0230/Positive/Ongoing]

### Implications for Future Pregnancies

Families who had terminated pregnancies and those with ongoing pregnancies both wanted to know if sequencing might provide information regarding future pregnancies.

MOTHER: Well I just wanted to, you know, learn why in some cases this happens and how does it—like—affected our future kids, if we planned on having any [0011/Negative Result/Termination]

MOTHER: I guess, what we were hoping is to see if there is anything else that we should be aware of in the future for us or for the baby. And so that is what we were kind of hoping to get out of it. [0565/Negative Result/Ongoing]

One specific reason for having sequencing was to inform IVF, as seen below,

MOTHER: We already have two more embryos frozen. So if there was any risk to those embryos, we wanted to know before we implanted them. So for us it is important to see if there was some genetic cause [0442/Negative/Terminated]

### Knowledge Is Powerful but Could Create Uncertainty

Finally, the theme of whether knowing is better than not knowing was reflected up on by some interviewees. In the following instance, the mother highlighted that they felt that the knowledge gained from sequencing could be powerful,

MOTHER: I wanted to know if anything was wrong. I believe knowledge is power—so I did not have any fears about it. [0398/Positive/Ongoing]

For others their hopes for sequencing were mixed with a certain degree of concern that knowing more might create more uncertainty. In the following example the mother highlighted that they might see knowledge as troubling and potentially worth avoiding, while their husband felt differently,

MOTHER: Well he [father] was. He’s more for like, you know, knowing everything, you know, information is power. Me, I am more of a, you know, ignorance is bliss. So I think I would have been okay without knowing too much. [0260/Positive/Ongoing]

Finally, in the following instance the interviewee had initially turned down sequencing, but then decided to have sequencing based on not wanting to miss out on potentially important information sooner rather than later. The mother was also explicit in saying that at-first she did not want to do the exome test as she was worried that it would add an even greater strain (greater than the early tests showing problems with the fetus).

MOTHER: My immediate reaction was no; we should not do this. I thought it was going to bring up more questions than answers … I was really worried that we were going to get more uncertain answers that would be even harder to make a decision on because we would know there was something but we would not know what it meant … I think we just wanted to know—I guess really, I was hoping to learn that there was nothing wrong, but if there was something wrong, I wanted to know sooner rather than later. [0309/Positive Result/ Ongoing]

## Assessment of Value

### Identifying Cause

Arguably the simplest form of evaluative framework was in respect to a positive result providing knowledge of the cause of fetal demise,

They were able to pinpoint, you know, what happened and what was the gene that caused it and, you know, that’s all we could ask for really, just finding exactly what happened… They knew there was a variant, but they could not pinpoint what it was, so I think this test definitely gave us the information that we needed. [0195/Positive Result/Deceased after birth]

However, the benefits of identifying causality might be interpreted in more nuanced and sometimes ambiguous manners. For example, a negative result might be seen as beneficial because the alternative—finding a genetic cause—might present a worst-case scenario. The fear of finding a genetic cause may be linked to the fear of being culpable for passing on a variant (as discussed below),

MOTHER: Like, nobody told us, “We guarantee there is no genetic link”. However, what we did hear is, “Based on everything that we know today, there was no genetic link,” essentially. And so, I think that is the best news that we could have hoped for. I think the only way that we would have gotten the clarity that we would have liked to hear is if they did find a link, and that would have been bad news. 0348/Negative/Terminated]

Not finding an identifiable cause might also be seen beneficial to families in the reassurance provided that there was nothing that could have been done (again, possibly suggesting that if a genetic cause had been identified it may have indicated a degree of parental culpability in passing on the variant),

INTERVIEWER: Do you still think about the results you received … is it something that is on your mind?

MOTHER: If it was genetic, I guess there would be something we could almost tangibly do with it and be like, “Okay, there is something wrong with one of us.” But it was not. So we think of it in the fact that again, there is nothing we can do. So it was just very unfortunate. So I guess that when I look back at everything that happened, that is how I look at it, is there was nothing we could have done to prevent this. [0442/Negative/Terminated]

This sense of relief that those families were not responsible for the fetal anomaly was also seen in positive cases that were *de Novo* (not inherited), as seen below,

FATHER: It’s nobody’s fault, it’s something they do not know why it happened. That is the purpose of the tests, to clarify. And it’s very helpful because it’s easy to say, “No, it’s your fault.” But the tests have clarified all that and they explained it very well to us. That’s the purpose of the test, to clarify. [0230/Positive/Ongoing]

Finally, it was notable that having a positive result was not always seen as the end of the story—especially when there was some ambiguity in the interpretation. It was still something to hold onto, in the hope that science might catch up one day,

MOTHER: So, I am happy thereis like something to hold onto. There is a name, and maybe it did not fit the complete profile, but maybe that profile will even change over time as more of these occur. And then, maybe like in a few years, I will even know that oh yeah, for sure, that was that it—the thing. [0041/Positive/Terminated]

### Implications for Current Pregnancy and Postnatal Care

A positive sequencing result might be seen as helpful in planning for the future, as seen in the following instance,

MOTHER: And the defect had a name so, you know, it was not anything out of the ordinary. So, you know, there was a solution. There was surgery. It was open-heart surgery but, you know, yeah, but it was something that could be fixed. [0260/Positive/Ongoing]

However, it was not always the case that a positive result clarified issues around current pregnancy and postnatal care. In the following instances, positive results left them with more questions than answers,

MOTHER: I was confused, I guess. It was nice to know, then some of the things that they said that were associated with this made sense when it came to me … but then it just made me super confused. I have this, “is there levels? Is there different degrees? Is there different severities? Is my daughter going to be just like me?” So it just made me have more questions. [0398/Positive/Ongoing]

MOTHER: All the information they gave us made it very hard for us to decide whether we would continue with the pregnancy or not. It put us between a rock and a hard place … I think we made the right decision, and it was not based on the genetic test. The test helped us to be prepared because we knew that she was going to be born with difficulties and all that. So, it helped us but it did not help us make the decision whether to have her or not. It is very hard to explain it and it’s very hard for people to understand. [0230/Positive/Ongoing]

Others noted that while cause might have been identified, the course of pregnancy management and postnatal care was not necessarily altered by the result, this might be the case due to uncertainty of result or even when the result is positive, as seen below,

MOTHER: All this is new so, as I said, when they told me I felt sad to know what could happen. However, I have more faith in God regarding how the baby will be born. Now, you can not see her. The ultrasounds are fine, the results of the heart ultrasound was normal, she moves, so, all we have to do is wait. [0596/Inconclusive/Ongoing]

MOTHER: I do not know that I felt any different because the confirmation that it was genetic, we did not change anything because nothing changed—you know, because the outcome does not change. It does not—that does not change. We are still having surgery, [0260/Positive/Ongoing].

### Implications for Identifying Carrier Status and Likely Outcomes in Future Pregnancy

Multiple interviewees highlighted the importance of prenatal sequencing for planning future pregnancies, including positive and negative results. What was of particular significance was identification of whether one or more parent is a carrier.

MOTHER: It was really reassuring to know that neither of us—both of us are not carriers—for this, you know, like a horrific genetic condition that our child seemed to be presenting with. [0086/Positive Result/Terminated]

Others reflected upon how it was useful to know the likely odds of having a child with the same condition in future,

MOTHER: So from that perspective, I guess this is the better result because it allows us to try again with some peace of mind that, again, no guarantees, but it’s a little bit easier to try again when you know that you are not facing 50% odds, or whatever was quoted initially. [0348/Negative Result/Terminated]

MOTHER: They had told us that there was nothing in our genetics that caused [Proband’s] heart condition…but they had said that there was like a 13% chance that my future child would have the heart condition as well, [0370/Negative Result/Ongoing]

However, inconclusive results might be seen as especially unhelpful for planning for future pregnancies given that such a result re-enforces uncertainty, as seen below,

So the result itself I feel like was rather unhelpful because I fell into the unfortunate bucket of, there is something but we do not know if it’s good or bad. So, it’s not enough to make decisions on [referring to IVF], it’s not definitive but it’s also not as reassuring. [0273/Inconclusive Result/Terminated]

Finally, in the one instance wherein sequencing appears to have a direct impact upon termination, the mother reported her feelings about how sequencing had changed her perception of pregnancy, perhaps increasing concerns and uncertainties about the fragility of life,

I think it just opened my eyes to all of the things that can possibly go wrong. I think this was just one little, tiny thing that went wrong in one gene out of, I do not know how many genes you have, thousands and thousands of genes. And it’s like, that could happen anytime. And it’s really amazing that so many children are born normal … I worry about future pregnancies, probably more than I would have. But I, if you think about it, if we would have had that kid, I would have been even just as worried because of all the issues that the kid would have had. [0309/Positive Result/Ongoing]

### The Value of Knowledge

While increased or perhaps continued uncertainty was a significant element for some families in how they might interpret results, for others having genomic sequencing provided knowledge that they otherwise would not have had, and thus the process itself was valued. This was seen with positive and negative results, as seen below,

MOTHER: I feel grateful to have some more information that we otherwise would not have had [0086/Positive/Terminated].

MOTHER: I am like—really happy and like thankful that we actually went through this and like, you know, just learn a little more about it. [0011/Negative/Terminated]

FATHER: I think, at least, it gives us choices, and where knowledge is better than not having nothing. [0309/Positive Result/ Ongoing]

MOTHER: I feel reassured because I feel like we have done what we can to really find out about the baby’s condition and with this additional study, it just kind of provides us even more information and being just educated. I feel like it’s one less thing that we have to worry about for the baby, I guess? [0565/Negative/Ongoing]

## Discussion

The hopes expressed by families in our study are similar to those found in non-prenatal contexts; they include a hope that sequencing can find cause, guide treatment, and predict risk and family planning (Khan et al., 2016; Wynn et al., 2018; Donohue et al., 2021). Our results suggest that in evaluating the benefits of prenatal genomic sequencing, the families in this study construed benefits in a variety of ways that allow for considerable flexibility of interpretation. In general, this flexibility of interpretation enabled them to infer largely positive evaluations of the experience of genomic sequencing; again, reflecting findings outside of the prenatal context (Biesecker et al., 2014; Stivers and Timmermans, 2017; Robinson et al., 2019; Mollison et al., 2020; Donohue et al., 2021).

Despite the presence of continued uncertainty in respect to treatment, care, and family planning options, families in the study largely valued the opportunity to have greater etiological knowledge. This was true for families with ongoing and terminated pregnancies. Positive results were seen to provide some degree of closure through knowledge of the cause of fetal demise and/or the potential that more would be known in the future about the cause of this and other forms of fetal demise. In respect to ongoing pregnancies, positive results were seen to provide a degree of foresight; even if this foresight included likely hospitalization and knowledge that the variant did not provide clear clinical interventions. A positive result allowed families to move forward, or at least have some insight into what lay ahead for them. Negative results in ongoing and terminated pregnancies were viewed as helpful in two instances. Firstly, that everything had been done to find out the cause of fetal anomalies. Secondly, that a negative result suggested that future pregnancies are unlikely to be impacted (as the condition was not genetically inherited). The latter interpretation may be tied to a broader sense of relief among the families interviewed of being absolved of guilt for passing on a deleterious genetic variant. Overall, negative and positive *de novo* results (which accounted for five out the six positive cases) results may have allowed families to move on in their lives after sequencing without feeling guilty for passing on a genetic variant. The importance of guilt and absolution from guilt in passing on a deleterious genetic variant is also seen in families in a pediatric setting (Stivers and Timmermans 2017; Malek et al., 2019; Mollison et al., 2020). Finally, several interviewees reflected upon how undertaking sequencing allowed them to meet a sense of obligation to do something. Again, this sense of having or wanting to do something is found in literature on pediatric exome sequencing for rare conditions, and in respect to how sequencing offers an opportunity to be pro-active in trying to at least find out more about a condition or set of symptoms (Malek et al., 2019; Luksic et al., 2020; Mollison et al., 2020; Donohue et al., 2021).

This generally positive overview of the value of sequencing to families must be seen against the notable limitations of sequencing to identify treatment or care options (or identify the best family planning options). At times family hopes were not fulfilled. This was largely seen in terms of how families described the ambivalence of results in respect to treatment or care options. This ambivalence was especially evident with an uncertain result, but even with positive results, prenatal genomic sequencing was sometimes perceived by families in the study to add uncertainty as to the course of treatment or where to go from this point onwards.

Unfortunately, due to the size of the population and study intake, the degree to which genomic sequencing plays a role in the termination of pregnancy was difficult to interpret. In this study—as with the study by Wou et al. (2018) in a vast majority of cases the decision to terminate was based upon prior findings of fetal anomalies. In the one instance wherein sequencing played a major role in termination, there was still a sense that it was better to know than not know. However, it is also the case that at least two families reflected upon (hypothetically) how genomic sequencing could provide information that may force them into making the difficult decision about whether to continue a pregnancy; a decision that they might otherwise avoid having to make if the information was not available to them. This nuanced, individualized, and non-deterministic view of sequencing in respect to pregnancy termination is similar to the findings of other literature (Kalynchuk et al., 2015; Richardson and Ormond, 2018). However, any such conclusions about pregnancy decision making are highly tentative given the size of the study.

Families in this study appeared be attuned to dealing with the uncertainties that may arise from receiving results of any kind. As our study strongly suggests, families believed that genomic sequencing could help them in their diagnostic journey and were prepared for the uncertainties and limitations that are inherent to sequencing. In reflecting upon how this study might inform genetic counselling, it is notable that while families seemed able to navigate many of the uncertainties of prenatal genomic sequencing, their relative success may well be a function of the extended counselling sessions observed. Ultimately, it is essential that genomic counsellors (among others) prepare families for these uncertainties and guide families through their respective results (Yurkiewicz et al., 2014; Harris et al., 2018; Mellis et al., 2018; Ferretti et al., 2019; Lewis et al., 2021; Talati et al., 2021).

### Limitations/Further Studies

Our sample size of 15 families means that subdivisions into representative groups by result and by pregnancy context allow for only limited examples of each group. Further research with larger samples is needed to see whether these findings can be replicated through surveys and interview-based studies. Nevertheless, it is argued that there is sufficient distinction to warrant highlighting the differences and similarities in expectations and assessments of the benefits and limitations of genomic sequencing in a prenatal context. It should also be noted that these conditions were rare, making these interpretations perhaps somewhat distinct from more commonly seen genetic variants or fetal anomalies. It is also notable that in the vast majority of interviews the decision whether to continue the pregnancy was taken prior to genomic sequencing, making it difficult to interpret the role of sequencing on termination decisions. Finally, this study was too small to explore cultural differences in attitudes to prenatal genomic testing (Chen et al., 2013; Tsai et al., 2017)

## Conclusion

Although our sample is small, it suggests that families may be willing to live with the uncertainties presented by prenatal genomic sequencing pre- and post-results and are potentially able to benefit through the knowledge gained through sequencing. This may well be a function or indicator of the success of genetic counsellors in guiding families through the process of genomic sequencing. We have noted that uncertainties are likely to remain a strong feature of prenatal genomic sequencing for a considerable period. Our data suggest that families may be willing to live with this uncertainty for the present, but that support structures are essential to guide them through their expectations and interpretations of results. Finally, one should not underestimate the importance to families of simply trying to do something to gain knowledge, and the inherent value of sequencing in meeting the desire to try anything to reduce uncertainty.

## Data Availability

The datasets presented in this article are not readily available because the study includes qualitative interview data. I can confirm that I [SO] have full access to all the data in the study and responsibility for the integrity of the data and the accuracy of the data analysis. The datasets from which excerpts are presented in this article are not readily available as a condition of the study is that raw data will not be shared outside of the research team. Upon request, sections of the data may be provided for specific research requests after the permissions of participants are requested and received. Requests to access the datasets should be directed to simon.outram@ucsf.edu.
